# Interventions to support mental health in people with long COVID: a scoping review

**DOI:** 10.1186/s12889-023-16079-8

**Published:** 2023-06-20

**Authors:** Hiyam Al-Jabr, Lisa D. Hawke, David R. Thompson, Andrew Clifton, Mark Shenton, David J. Castle, Chantal F. Ski

**Affiliations:** 1 Integrated Care Academy, University of Suffolk, Ipswich, UK; 2 Centre for Addiction and Mental Health, Toronto, Canada; 3 School of Nursing and Midwifery, Queen’s University Belfast, Belfast, UK; 4 Department of Psychiatry, University of Toronto, Toronto, Canada

**Keywords:** Long COVID, Mental health, Optimal Health Programme, COVID-19, Integrated care

## Abstract

**Introduction:**

Long COVID (LC) is a multisystem disease with symptoms lasting weeks or months beyond the acute COVID-19 infection. Several manifestations are reported by people with LC, including effects on mental health, with varying degrees of psychological distress and disturbances to daily activities. Research conducted to identify effective interventions to support mental health among people with LC has been limited by the breadth and scope of studies.

**Aim:**

This review aims to identify interventions being tested to support mental health of people with LC.

**Methods:**

A scoping review was conducted by searching five databases for articles published between January 2020 and early October 2022 to identify research evaluating interventions focused on improving mental health symptoms associated with LC. Results from all sources were checked for eligibility by two reviewers, and agreements were resolved by discussion. Gray literature and reference list of included studies and relevant reviews were scrutinised to identify any additional studies. Data extraction was conducted by one reviewer and checked by another reviewer for accuracy.

**Results:**

Of the 940 studies identified, 17 were included, the design of which varied but included mainly case studies (*n* = 6) and clinical trials (*n* = 5). Several interventions were described, ranging from single interventions (e.g., pharmacologic) to more holistic, comprehensive suites of services (pharmacologic and non-pharmacologic). Several mental health outcomes were measured, mostly anxiety and depression. All included studies were reported to be associated with improvements in participants’ mental health outcomes.

**Conclusion:**

This scoping review identified studies reporting on a variety of interventions to support mental health among people with LC. Although positive changes were reported by all studies, some were case studies and thus their findings must be interpreted with caution. There is a need for more research to be conducted to identify the impact of interventions on mental health of people with LC.

**Supplementary Information:**

The online version contains supplementary material available at 10.1186/s12889-023-16079-8.

## Introduction

The COVID-19 pandemic began in China in December 2019 [1], and as of December 2022, it had claimed the lives of more than six million people and infected more than 640 million around the world [2]. Most attention has focused on the acute COVID-19 infection [3], with comparatively little devoted to the longer term and ongoing symptoms experienced and reported by many people [4]. These people, who often felt their symptoms were neglected or disbelieved, formed support groups on social media, drawing global attention to this ongoing condition, which was named ‘long COVID’ (LC) [5]. This condition has been described by several other labels, including post-COVID, long haulers, post-acute COVID, and post-SARS-COV-2 [6–8]. It has also several definitions [9]. For example, in the UK, according to the National Institute for Health and Care Excellence (NICE), LC represents “*signs and symptoms that develop during or after an infection consistent with COVID-19, continue for more than 12 weeks and are not explained by an alternative diagnosis… It includes both ongoing symptomatic COVID-19 (from 4 to 12 weeks) and post-COVID-19 syndrome (12 weeks or more)*” [10]. Similarly, the World Health Organisation (WHO) defines LC as follows: “*Post-COVID-19 condition occurs in individuals with a history of probable or confirmed SARS-CoV-2 infection, usually 3 months from the onset of COVID-19 with symptoms that last for at least 2 months and cannot be explained by an alternative diagnosis*.” [11].

Research on LC shows that following the acute viral infection, damage can occur in different organs in the body including the brain [12]. There are multiple potential symptomatic sequelae including musculoskeletal problems, cognitive impairment, fatigue, and psychological distress [13–16].

Chronic illness and disability caused by LC represents a major emerging health challenge [4], LC can affect all aspects of an individual’s daily life, including quality of life (QoL), social functioning, and ability to participate in normal activities [17, 18]. In addition, mental health problems appear common in LC [19–22].

The precise proportion of people who contract COVID-19 and have LC has not been firmly established [23–25], though it is estimated that, globally, around 43% of people experience some degree of long-term symptoms following the acute infection [26]. In the UK it has been estimated that more than 1.8 million people have LC [27], though this will likely increase as COVID-19 continues to circulate [3]. Those at most risk of LC are women, elderly people, smokers, obese or overweight people, and those living in socio-economically challenged areas [4, 28].

The impact of LC on mental health has been examined. Studies report that more than 87% of people with LC have mental health symptoms, including anxiety, depression, and cognitive problems [29–36]. As LC is a relatively new illness, it is surrounded by a great deal of uncertainty: it can manifest differently from one person to another, with more than 200 associated symptoms [31] and presentations that do not necessarily fit into established clinical categories [4]. Indeed, some consider LC to be a form of post-viral syndrome [37], which provides some evidence base for treatment and management. However, in the face of uncertainty, patients can be at risk of being either over-investigated and over-treated, or under-investigated and under-treated [4]. As such, no evidence-based treatment options are yet available [38–41]. Usual care currently provided to people with LC is through LC clinics and services, and general medical practices [42, 43].

A recent systematic review showed that while some research is currently being conducted to examine interventions to support the mental health of people with LC, the breadth and scope of this research is limited [35]. This systematic review was focused on ongoing trials that were conducted between January 2020 to May 2022. In this context, this scoping review aims to more broadly identify what interventions have been examined to support the mental health of people with LC.

## Methods

### Database search

This scoping review was conducted following Arksey and O’Malley framework [44] and is reported following PRISMA-ScR (Preferred Reporting Items for Systematic Reviews and Meta-Analyses Extension for Scoping Reviews) guidelines [45]. No priori protocol to this scoping review was published or registered. A systematic search was conducted on 3 October 2022 to identify relevant studies using MEDLINE, EMBASE, Web of Science, CINAHL, and PsycInfo databases from January 2020 to early October 2022. Searches were limited to journal articles. Records were exported initially into the reference manager Endnote, where duplicates were identified and removed, and then to Covidence software for data management [46].

### Search terms

Between 16 September and 3 October 2022, preliminary searches were initially conducted using the databases mentioned to identify appropriate keywords to use in this review. Search terms were selected based on the target population and the target intervention, and terms were grouped under three headings related to LC, intervention, and mental health problems associated with LC. The search keywords are detailed in Table 1, (see Additional file 1 for an example of the search strategy). Moreover, reference lists of included studies and related reviews were also examined to identify additional potentially eligible studies. Grey literature was also checked to identify any additional relevant studies, using the following resources: Social Care Institute for Excellence (SCIE), Canadian Agency for Drug and Technologies in Health (CADTH), King’s Fund, Open Grey, and the Health Foundation. The search strategy was adapted appropriately when searching the different databases.

**Table 1:** Search keywords

**Keyword heading**	**Keywords**
Intervention	intervention* or treatment* or support* or therap* or service or program*
**AND**	
Mental health	mental* or psychiatr* or "post-trauma*" or posttrauma* or PTSD or depress* or anxiet* or dysthymi* or phobia* or panic* or psychopath*
**AND**	
Long COVID	"long COVID" or "long covid*" or longcovid or "long-COVID" or "post COVID" or "post-acute COVID" or "post acute COVID" or "long haul*" or "COVID sequelae" or "sequelae of COVID" or "post-SARS-COV-2" or PASC or "Post-acute Sequelae of COVID-19"

### Study inclusion criteria

As is conventional with scoping reviews [47, 48], our inclusion criteria were deliberately broad, namely:

1. Research focused on LC and mental health
2. Research that introduces/discusses an intervention/treatment/support for mental health issues associated with LC
3. Research where assessment of mental health is the primary outcome (since mental health is commonly affected by LC and is also impacted by physical symptoms of LC).
4. Patient population is aged 18 years and above
5. Publications from 2020 to 2022
6. Original research (article/journal article) (including qualitative, quantitative, or mixed research methods, case reports or other study designs that reports an original research).

Studies that did not meet one or more of the above criteria or that met any of the following exclusion criteria were excluded from the review. Research that is focused on:

1. COVID-19 acute infection and/or its treatment
2. Treatment of mental health issues not related to/ induced by LC
3. Treating issues caused by LC but not related to mental health (e.g., respiratory, or cardiovascular problems).

### Study selection

Title and abstracts, then full text screening were independently carried out by two reviewers (HA and LDH), to assess article eligibility against the inclusion and exclusion criteria. Any arising disagreements were resolved by discussion between the two reviewers, and if necessary, by consulting with a third reviewer (CFS).

For studies not written in the English language but were found relevant at title and abstract screening, corresponding authors were contacted to request relevant information; when additional information was not provided by the authors, Yandex website (https://translate.yandex.com/doc) was used to translate these studies to confirm eligibility and extract relevant data. Additionally, where full texts of included studies were not retained even after contacting corresponding authors, their abstracts were included and used for data extraction.

### Data extraction

A data extraction template was designed using Microsoft Excel to extract the following items-where available- from each study:

1. Author(s) and publication year
2. Study design
3. Study setting and country
4. Participant information (recruited participants, age, gender, and ethnicity)
5. Practitioners involved in delivering the intervention/treatment
6. Description of the intervention
7. Description of the outcome measures and study findings.

Data extraction was carried out by HA and was reviewed and verified by LDH, AC, MS, DC, DRT and CFS to ensure accuracy and completeness. Disagreements were resolved by discussion or through consultation with a third reviewer (CFS).

### Data synthesis

A narrative approach was used to synthesize and describe the data extracted from included studies [49].

### Dealing with missing data

Corresponding authors were contacted by email to collect any missing data. When no response was received, studies were still included in the review with missing data designated as ‘not reported’.

## Results

**Fig. 1:**
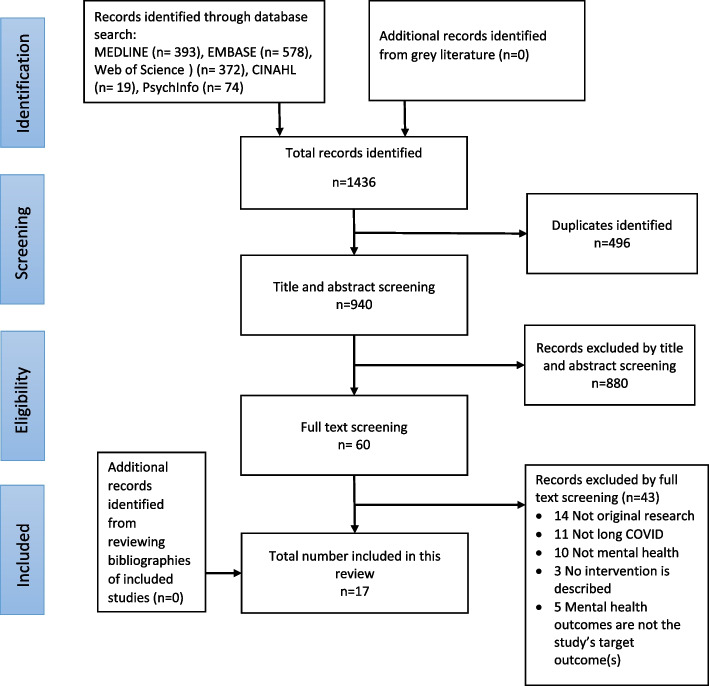
PRISMA flowchart

A total of 940 unique records were identified through the database search. No additional studies were identified from the grey literature or from the reference lists of included studies. After the complete screening and selection process, the final review encompassed 17 studies. The findings of the search process are presented in the PRISMA flow diagram (Fig. 1).

### General description of included studies

Table 2 provides an overview of included studies. Of all included studies, one study was retained based on a relevant abstract in the absence of a full text despite multiple attempts to obtain it [50]. All studies were written in the English language except for three, written in Russian [50–52]. The included studies were conducted in nine countries, with more than half conducted in Europe (*n* = 11, 64.7%), five of which were from the UK.

**Table 2:** Description of included studies

**Study, year [ref], location**	**Study design**	**Study setting**	**Recruitment method**	**Recruited participants**	**LC stage when recruited**
1. Brodsky, 2021 [53], USA	Case study with pre-post survey	Outpatient subspecialty clinic within a Healthcare System	Referral through medical doctors or self-referred. The program was extensively covered in TV and print media	***N*** = 12 pre-post, *n* = 2 Case study **Gender**: women **Age**: Mean 50 years; case study 50, 78 (mean 64) years **Ethnicity**: case study: White non-Hispanic	Not collected
2. Bogolepova et al., 2021 [51], Russia	Two arm clinical trial with pre-post assessment	NR	NR	***N*** = 100 **Gender**: 41 women, 59 men **Age**: range 22–71 (mean 50) years **Ethnicity**: NR	Had acute COVID-19 infection 5.4 months earlier
3. Tereshin et al., 2021 [52], Russia	Controlled trial	Secondary care	NR	***N*** = 45 (21 intervention and 24 control) **Gender**: 22 women, 23 men **Age**: Intervention group: mean 56 years Comparison group: mean 57 years **Ethnicity**: NR	NR
4. Fowler-Davis et al., 2021[54], UK	Qualitative and pre-post assessment	University setting in collaboration with a local GP collaborative group	Through a local community wellbeing group and social prescribing network	***N*** = 10 recruited; 8 completed **Gender**: 6 women, 2 men **Age**: range 38–73 years **Ethnicity**: white British (*n* = 4), others (*n* = 4)	At least 12 weeks post-infection (range 4–14 months)
5. Harenwall et al., 2021 [55], UK	Single arm pre-post assessment	Primary care	Self-registration to the programme (health, social and care staff, and outreach to key stakeholders of different sites, e.g., hospital, care home, police, council workers, teachers, local charity workers)	***N*** = 219 registered; 149 completed pre-course assessment; 76 completed post course assessment **Age**: Pre-course: mean 47 years; Post course: mean 49 years **Gender**: Pre-course assessment: 112 women, 16 men, 21 missing gender Post-course assessment: 53 women, 8 men, 15 missing gender **Ethnicity**: Pre-course: white British (67%), South Asian (13%), Asian British (4%), mixed heritage (3%), missing (13%). Post-course: White British (79%), South Asian (7%), Asian British (3%), mixed heritage (4%), undisclosed (7%)	A mean of 5.99 months since acute COVID-19 infection (SD 3.89)
6. Łuckoś et al., 2021 [56], Poland	Case study	NR	NR	***N*** = 1 **Gender**: woman **Age**: 48 years **Ethnicity**: NR	Had acute COVID-19 infection six months earlier
7. Compagno et al., 2022 [57], Italy	Single arm pre-post design	Hospital out-patient care	NR	***N*** = 30 **Gender**: 12 women, 18 men **Age**: mean 58 years **Ethnicity**: NR	A mean of 3 (range 1–6) months since resolution of acute COVID-19 infection
8. Esin et al., 2022 [50], Russia	Three arm clinical trial with pre-post assessment	Home or hospital setting	NR	***N*** = 92 **Gender**: NR **Age**: Group 1 (*n* = 40) mean 43 years Group 2 (*n* = 32) mean 45 years Group 3 (n = 20) mean 45 years **Ethnicity**: NR	Had acute COVID-19 infection 12 weeks earlier
9. Brough et al., 2022 [58], UK	Service evaluation with pre-post assessment	Community setting	Self-referral, advertised through social media, social prescribing network and word of mouth	***N*** = 25 **Gender**: 15 women, 7 men, 3 gender not disclosed **Age**: range 15–92 years **Ethnicity**: white British (*n* = 18), white Asian (*n* = 1), other white background (*n* = 2), Chinese (*n* = 1), undisclosed (*n* = 3)	NR
10. Wagner et al., 2022 [59], Austria	Case report	Hospital rehabilitation department	NR	***N*** = 1 **Gender**: woman **Age**: 55 years **Ethnicity**: NR	Had acute COVID-19 infection 6.5 months earlier
11. Garcia-Molina et al., 2022 [60], Spain	RCT	Secondary care	Eligible clinical participants were included without seeking their permission as the sample was derived from clinical practice (without altering the care protocols)	***N*** = 91 treatment group (and 32 control group) **Gender**: 55 women, 36 men in treatment group **Age**: mean 50 years in treatment group **Ethnicity**: Spanish, white	At least 12 weeks post-acute COVID-19
12. Philip et al., 2022 [61], UK	Parallel-group, single-blind RCT	Secondary care	Referral from LC clinics	***N*** = 150 (74 intervention, 76 control) **Gender**: 121 women, 26 men **Age**: mean 49 years **Ethnicity**: Intervention (white 82%, black/ British black 1%, Asian/British Asian 5%, mixed/multiple 4%, other 5%, undisclosed 3% Control: white (82%), black/British black (9%), Asian/British Asian (4%), mixed/multiple (3%), other (1%), undisclosed (1%)	A mean time of 320 days (around 10.5 months) since Acute COVID-19 infection
13. Tobinick et al., 2022 [62], USA	Case study	Secondary care	Treatment performed as part of standard medical practice, as requested by patient	***N*** = 1 **Gender**: woman **Age**: 48 years **Ethnicity**: White, non-Latino	Had acute COVID-19 infection 12 months earlier
14. Wang et al., 2022 [63], USA	Case study	NR	NR	***N*** = 1 **Gender**: woman **Age**: 55–60 **Ethnicity**: NR	Had acute COVID-19 infection over a year earlier
15. Skilbeck, 2022 [64], UK	Case study	Primary care	NR	***N*** = 1 **Gender**: man **Age**: 36 years **Ethnicity**: Asian British	Had acute COVID-19 infection 8 months earlier
16. Orendáčová et al., 2022 [65], Czech Republic	Single arm pre-post pilot study	Described as both secondary and quaternary care setting	Through social media	***N*** = 10 **Gender**: 7 women, 3 men **Age**: range 19- 46 (median 21) years **Ethnicity**: All participants are from Czech Republic	A median time of 12 months (range 3–19 months) since having acute COVID-19 infection
17. Koliadenko et al., 2022 [66], Ukraine	Single arm pre-post-follow up evaluation	Remote medical consultative psychological and psychiatric help	Self-referred for treatment	***N*** = 129 **Gender**: 68 women, 61 men **Age**: 27.9% between 50–59 years **Ethnicity**: NR	NR

*LC* Long COVID, *NR* not reported, *RCT* Randomised Controlled Trial

Of the 17 included studies, six were case studies [53, 56, 59, 62–64] and five were clinical trials [50–52, 60, 61], two of the latter were randomised controlled trials (RCTs) [60, 61]. Other designs encompassed four single arm pre-post studies [55, 57, 65, 66], one qualitative study [54] and a single service evaluation study [58]. Several settings were described by included studies including primary and secondary healthcare settings, a university setting in collaboration with a local GP collaboration group, outpatient subspeciality clinic, and hospital outpatient care. Three studies did not clearly report their setting [51, 56, 63].

Eight studies did not report the method used in recruiting participants [50–52, 56, 57, 59, 63, 64]. Described recruitment approaches included social media, referral from LC clinics, medical doctors, or a local community network. One study recruited staff with LC opportunistically from specific settings [55].

A total of 940 participants were recruited across all included studies, with individual study numbers ranging from one [56, 59, 62–64] to 219 [55]. All case studies (*n* = 6) recruited only one or two participants [53, 56, 59, 62–64]. Participants were recruited at different stages of their LC journey (i.e., at different time points since having the acute COVID-19 infection). This was reported by 13 studies [50, 51, 54–57, 59–65], ranging from one month post-acute COVID-19 [57] to over a year post infection [64, 65].

The gender of included participants was reported by all studies except one [50], but in two further studies, gender was reported as a missing variable for some participants [55, 58]. Most recruited participants were women (total *n* = 353, range 1–121) [51–54, 56–66, 63]. One study reported the gender of participants pre and post assessment [55].

Age of included participants was reported by all studies, either as a precise age (*n* = 5) [53, 56, 59, 62, 64], or as an average (mean, median) of all participants (*n* = 2) [57, 61], or as an average age of groups of participants allocated at different treatment groups or at different assessment time points (*n* = 4) [50, 52, 55, 60], or as a range (*n* = 6) [51, 54, 58, 63, 65, 66]. Across all studies, age ranged from a 15–24 age group to 92 years [58].

Ethnicity or cultural background of participants was reported for only nine studies, with half of these including only people identifying as white or Asian and the rest reporting a variety of ethnicities/cultural backgrounds.

### Description of study intervention/treatment

Table 3 describes the various interventions tested by the included studies, ranging from a single intervention (e.g., using a pharmacological product, sessions of electromagnetic field, modifications to diet) (*n* = 7) [50, 51, 59, 61–63, 65], to integrated programmes covering diverse elements of health (*n* = 10) [52–58, 60, 64, 66]. Healthcare components included rehabilitation programmes, lifestyle interventions, stress management, sleep hygiene, breathing techniques, optimising diet, energy conservation, psychoeducation, maintaining social contacts with close networks, reading, relaxation techniques and cognitive behavioural therapy (CBT). Some interventions were focused on testing the effects of certain pharmacological products or nutritional changes on enhancing outcomes.

**Table 3:** Description of interventions/treatment

**Study**	**Intervention**	**Description of the intervention**	**Practitioners delivering the intervention**	**Duration and intensity of intervention delivery**
1. Brodsky, 2021 [53]	Post-COVID-19 myalgic-encephalomyelitis (post-COVID-19 ME) program	Multiple components related to diet, pain management, breathing, and lifestyle interventions (e.g., walking, sleep hygiene, and CBT)	Medical doctors	Weekly for 4 weeks (with certain aspects, e.g., walking, diet and sleep, practised daily)
2. Bogolepova et al., 2021 [51]	Cholytilin and MexiB 6	Two study groups: participants with anxiety/depression dominance received MexiB 6; participants with cognitive impairment dominance received Cholytilin	NR	MexiB 6: One tablet 3 × day Cholytilin: 2 capsules (800 mg) AM, 1 capsule (400 mg) PM Follow up period was 60 days
3. Tereshin et al., 2021 [52],	Intravenous Cytoflavin in combination with a standard rehabilitation program	Control group received a standard post-COVID rehabilitation programme (included pulse magnetic therapy, inhalation therapy, aeroion therapy, aerobic training, psychotherapy, nutritional supplementation, and standard drug therapy). In addition to standard care, intervention group received intravenous Cytoflavin	Cytoflavin: NR Rehabilitation program: some aspects NR, however included a medical psychologist and psychotherapist for psychotherapy component	Cytoflavin: Daily for 10 days Rehabilitation program: 21 ± 2 days
4. Fowler-Davis et al., 2021 [54],	Rehabilitation intervention (LC support sessions)	A co-produced virtual ‘clinic’ delivered by a team of practitioners who met participants three times each to directly consider their needs and offer structured advice	Sport and exercise medicine doctor, an occupational therapist, clinical psychologist or physiotherapist	Three sessions delivered over 3–6 weeks (first two sessions delivered over consecutive weeks and third sessions delivered 2–3 weeks later)
5. Harenwall et al., 2021 [55],	Primary Care Wellbeing Service (PCWBS) “Recovering from COVID” rehabilitation course	Virtually delivered psychoeducation and support that focused on enhancing several health areas affected by LC, including sleep, diet, voice, activity, energy, stress management, breathing, and relapse planning	A clinical psychologist, physiotherapist, occupational therapist, dietitian, speech and language therapist, assistant psychologist, and a personal support navigator	One 60 min virtual session per week for 7 consecutive weeks
6. Łuckoś et al., 2021 [56]	Neurotherapy	Two treatment programmes: administration of neurofeedback (programme A); goal-oriented cognitive training (programme B), which included various strategies (e.g., organising sleep, exercise, healthy diet, maintaining social contacts)	Neuropsychologist, neurorehabilitation, locomotor disorder clinic	Twice a week for 15 weeks (both treatments)
7. Compagno et al., 2022 [57]	Out-of-hospital multidisciplinary rehabilitation (MDR) program	Assessment, physical training, tailored psychosocial treatment (based on cognitive behavioral therapy, eye movement desensitization and reprocessing therapy, with individual relaxation techniques and, multidisciplinary counselling for lifestyle modification)	Multidisciplinary team including physical trainers, nurses, cardiologists, psychologists, and sport medicine physicians	Physical training: 3 90-min sessions/week, range 8–20 sessions; Psychological treatment: 4 sessions
8. Esin et al., 2022 [50]	Anvifen (GABAergic nootropic drug with an anxiolytic effect)	Participants were divided into three groups: - Group 1: treated with Anvifen at home; - Group 2: treated with Anvifen in a hospital without oxygen support; - Group 3: treated with Anvifen in a hospital with oxygen support	NR	500 mg three times a day for 21 days
9. Brough et al., 2022 [58]	A package of care	Group practical and psychoeducational sessions with optional physiotherapy or Craniosacral Therapy (CST), after-care package (e.g., aromatherapy product, mindfulness coloring book, herbal tea, service information)	Health practitioner specialised in CST, a physiotherapist and other facilitators specialised in delivering other sessions	Group sessions: One per week for 4–6 weeks Physiotherapy: two 45 min sessions; CST: up to four 1 h sessions
10. Wagner et al., 2022 [59]	Pulsed electromagnetic field (PEMF)	PEMF treatment using a certified therapy device	NR	Twice weekly for 5 weeks
11. Garcia-Molina et al., 2022 [60]	Outpatient neuropsychological intervention programme	Respiratory therapy, physiotherapy, virtual neuropsychological rehabilitation (which included cognitive treatment), emotional intervention; artificial intelligence algorithms used to create a personalised plan based on the results of the pre-treatment evaluation	Clinical psychologists specialised in neuropsychology	Five sessions per week for 8 weeks (average of four sessions per week)
12. Philip et al., 2022 [61]	English National Opera (ENO) Breathe	A group online delivered breathing and wellbeing programme focused on breathing re-training through singing techniques and utilising lullabies, with access to online resources and regular email contact	LC clinic team including voice specialists	Once a week for 6 weeks (core group intervention), plus self-directed activities
13. Tobinick et al., 2022 [62]	Peri-spinal etanercept	Peri-spinal administration of etanercept	An internal medicine medical practitioner	Single 25 mg dose
14. Wang et al., 2022 [63]	High-fibre formula	Nutritional supplementation using a high fibre formula	NR	Gradual increase in high-fibre diet to three times a day for 2 months
15. Skilbeck, 2022 [64]	Cognitive Behavioural Therapy (CBT)	Virtual CBT sessions including psychoeducation, understanding current symptoms, normalisation and acceptance, goal setting, symptom monitoring, graded activity pacing, measuring patient progress, positive reinforcements, physical and psychological symptoms, reviewing goals, and moving forward	An accredited CBT therapist supervised by an accredited senior CBT therapist and long-term health conditions specialist	12 sessions over 5 months (Intensity NR)
16. Orendáčová et al., 2022 [65]	Neurofeedback (NFB)	Five sessions of NFB (included locating electrodes on the right and left temporal lobes)	A certified neurofeedback therapist	2 weeks: Two NFB sessions delivered at first week and three at the second week (25–45 min sessions)
17. Koliadenko et al., 2022 [66]	Virtual medical consultative psychological and psychiatric therapy	CBT protocols for generalized anxiety disorder and hypochondriacal disorder in combination with antidepressants and nonbenzodiazepine tranquilizer	NR	NR

Practitioners involved in delivering the intervention were reported by 12 studies, five of which engaged practitioners of a particular speciality, (e.g., medical doctors or clinical psychologists) [53, 60, 62, 64, 65]; one was being supervised by a cardiopulmonary specialist [64]. A multidisciplinary team approach was adopted in the other six studies, including physicians, occupational therapists, psychologists, physiotherapists, dieticians, speech and language therapists, sport medicine specialists and CBT therapists [54–58, 61]. The remaining six studies did not report who was involved in delivering the intervention [50–52, 59, 63, 66].

The duration of intervention delivery was reported by all studies except one [66], and ranged from a single dose [62] to 20 weeks [66]. Frequency of intervention delivery was not clearly described by two studies [64, 66], however, it varied widely between the remaining studies from daily [50–52, 63], once a week [53–55, 58, 61], twice a week [56, 59], or multiple times a week [57, 60, 65].

Some studies used a virtual approach to deliver the intervention (using online platforms for example to deliver sessions or a virtual clinic) [54, 55, 60, 61, 64, 66]. One study used an artificial intelligence-based intervention to deliver a personalised care approach [60].

### Description of study outcomes

Outcome measures are described in Table 4. Mental health outcomes included anxiety, depression, stress, sleep, neuropsychological functioning, and wellbeing. The timing of outcome measures was reported by all studies except one. Most studies used pre- and post-intervention measures, with additional follow-ups.

**Table 4:** Description of outcome measures and findings

**Study**	**Mental health-related outcome(s)**	**Questionnaire/assessment method used**	**Times of outcome measurement**
1. Brodsky, 2021 [53]	Health-related QoL	SF-12	Before and after 4 weeks intervention
2. Bogolepova et al., 2021 [51]	Anxiety, depression	HADS	Before and after 60 days of the intervention
3. Tereshin et al., 2021 [52]	Depression	HDRS	Before and at the end of the intervention
4. Fowler-Davis et al., 2021 [54]	QoL	EQ-5D	Before and after intervention
5. Harenwall et al., 2021 [55]	Health-related QoL General health	EQ-5D-5L Single item visual analog scale	Retrospective pre-Covid, before and after intervention
6. Łuckoś et al., 2021 [56]	Neuropsychological functioning	Standard Polish version of Mindstreams™ Interactive Computer Tests	Before and after intervention
7. Compagno et al., 2022 [57]	Health-related QoL, depression, anxiety	SF-36, Zung SRDS, Zung SRAS	Before and after intervention
8. Esin et al., 2022 [50]	Anxiety, depression, Health-related QoL and sleep	HADS, SF-36 FAS	Before and after intervention
9. Brough et al., 2022 [58]	Physical, mental, emotional, spiritual, social wellbeing	WHHQ-18	At the start of the first and last session of the intervention
10. Wagner et al., 2022 [59]	Anxiety, depression, health related QoL, perceived stress, resilience	SF-36, GAD-7, PHQ-9, PSS-10, BRS	Before, immediately after intervention and 6 weeks later
11. Garcia-Molina et al., 2022 [60]	Anxiety and depression	HADS	Before intervention, during last week of intervention and 6–7 months later
12. Philip et al., 2022 [61]	Health-related QoL	SF-36	At first week and at last week of intervention
13. Tobinick et al., 2022 [62]	Depression	BDI-II	Before and 24 h after intervention and 29 days later
14. Wang et al., 2022 [63]	Anxiety	Scale of 0 to 10	Daily
15. Skilbeck, 2022 [64]	QoL, depression, anxiety	COV19-QoL scale, PHQ-9, GAD-7	Before, immediately after the intervention and 3 months later
16. Orendáčová et al., 2022 [65]	Anxiety, depression	BAI, BDI-II	Before starting and after completing the intervention, and one week and one month later
17. Koliadenko et al., 2022 [66]	Depression, anxiety, stress wellbeing, activity, mood	DASS-21 Spielberger-Khanin scale of reactive and personal anxiety, WAM	At the initial visit, at the end of the intervention, and 3–6 months later

*BDI-II* Beck Depression Inventory-II, *BAI* Beck Anxiety Inventory, *BRS* Brief Resilience Scale, *DASS-21* Depression, Anxiety and Stress Scale-21, *EQ-5D-5L* Euro-Quality of Life-5D, *GAD-7* General Anxiety Disorder-7, *FAS* Fatigue Assessment Scale, *HADS* Hospital Anxiety and Depression Scale, *HDRS* Hamilton Depression Rating Scale, *PHQ-9* Patient Health Questionnaire, *PSS-10* Perceived Stress Scale-10, *SF-12* Short Form-12 items, *SF-36* Short Form-36, *WAM* Wellbeing, Activity and Mood questionnaire, *WHHQ-18* Warwick Holistic Health Questionnaire, *Zung SRDS* Self-Rating Depression Scale, *Zung SRAS* Self-Rating Anxiety Scale, *QoL* quality of life

The most commonly reported questionnaires for anxiety and depression were the Hospital Anxiety and Depression Scale (*n* = 3), the Generalized Anxiety Disorder-7 (*n* = 2) and the Patient Health Questionnaire-9 (*n* = 3). The Short-Form-36 questionnaire was mostly reported for measuring health-related QoL (*n* = 4). Other studies used a wide array of scales and measures, as detailed in Table 4.

### Description of study findings

Table 5  provides a description of study findings. Positive findings were reported by all studies. Nine studies reported significant improvements in different outcomes following the intervention [50–52, 55, 57, 60, 61, 63, 65], of which five are trials [50–52, 60, 61].

Some of the interventions used by these studies included using drug products i.e., Cholytilin and MexiB 6 for anxiety and depression, Intravenous Cytoflavin in combination with a standard rehabilitation program for depression, Anvifen for anxiety, depression, health-related QoL and sleep. Significant improvements were reported on these outcomes. Other studies examined wellbeing services or rehabilitation programs, such as Multidisciplinary Rehabilitation (MDR) for anxiety, depression and health-related QoL, and Primary Care Wellbeing Service (PCWBS) for general health and health-related QoL, with both showing significant improvement in these outcomes. Neuropsychological programmes included cognitive treatment, together with other respiratory and physiotherapy, for anxiety and depression, that were significantly improved. An English National Opera (ENO) programme that was delivered online and was focused on breathing re-training through singing techniques and utilising lullabies, was used to assess health-related QoL with significant improvement only reported to the mental health composite score. Another intervention included using a high-fibre formula examined anxiety that was reported to be significantly improved. Lastly, a neurofeedback (NFB) program that included locating electrodes on the right and left temporal lobes to examine anxiety and depression, with significant improvements reported. (See Tables 3, 4 and 5 for more details).

**Table 5:** Description of study findings

**Study, year [reference]**	**Intervention**	**Outcomes**	**Mental health-related findings**
1. Brodsky, 2021 [53]	Post-COVID-19 ME program	Health-related QoL	Positive effects reported on QoL for some patients; subjective improvements in work, social, home life
2. Bogolepova et al., 2021 [51]	Cholytilin and MexiB 6	Anxiety, depression	Significant improvements in both the MexiB 6 and Cholytilin groups: Anxiety, depression, and physical wellbeing
3. Tereshin et al., 2021 [52]	Intravenous Cytoflavin in combination with a standard rehabilitation program	Depression	Significant improvement: depression
4. Fowler-Davis et al., 2021 [54]	Rehabilitation intervention (LC support sessions)	QoL	Acceptable to participants; engaged and empowered participants to undertake healthy behaviour changes enhanced wellbeing
5. Harenwall et al., 2021 [55]	PCWBS	Health-related QoL General health	Significant improvement: general health, QoL
6. Łuckoś et al., 2021 [56]	Neurotherapy	Neuropsychological functioning	Positive effects reported on cognitive function domains, subjective improvement in sense of independence, memory, return to work
7. Compagno et al., 2022 [57]	MDR program	Health-related QoL, depression, anxiety	Significant improvements: physical and mental QoL domains, anxiety, depression
8. Esin et al., 2022 [50]	Anvifen (GABAergic nootropic drug with an anxiolytic effect)	Anxiety, depression, health-related QoL and sleep	Significant improvements: anxiety, depression, sleep, physical health QoL, mental health QoL
9. Brough et al., 2022 [58]	A package of care	Physical, mental, emotional, spiritual, social wellbeing	Positive effects reported on wellbeing for almost all participants; strong acceptability of the intervention
10. Wagner et al., 2022 [59]	PEMF	Anxiety, depression, health related QoL, perceived stress, resilience	Increased energy, QoL, resilience; decreased anxiety, depression, and stress. Subjective sense of being fully recovered, resumed work and private life, and improved wellbeing
11. Garcia-Molina et al., 2022 [60]	Outpatient neuropsychological intervention programme	Anxiety and depression	Significant improvements: anxiety, depression
12. Philip et al., 2022 [61]	ENO Breathe	Health-related QoL	Significant improvement: mental health QoL composite score No significant difference: physical health QoL composite score
13. Tobinick et al., 2022 [62]	Peri-spinal etanercept	Depression	Rapid improvements in depression and other non-mental health variables
14. Wang et al., 2022 [63]	High-fibre formula	Anxiety	Significant improvement: anxiety
15. Skilbeck, 2022 [64]	CBT	QoL, depression, anxiety	Positive effects reported on depression, anxiety, QoL
16. Orendáčová et al., 2022 [65]	NFB	Anxiety, depression	Significant improvements: anxiety and depression
17. Koliadenko et al., 2022 [66]	Virtual medical consultative psychological and psychiatric therapy	Depression, anxiety, stress wellbeing, activity, mood	Positive effects reported on depression, anxiety, stress

*CBT* Cognitive Behavioural Therapy, *ENO* English National Opera, *MDR* multidisciplinary rehabilitation, *ME* Myalgic-Encephalomyelitis, *NFB* Neurofeedback, *PCWBS* Primary Care Wellbeing Service, *PEMF* Pulsed electromagnetic field, *QoL* Quality of life, *LC* long COVID

## Discussion

### Summary of main results

To the best of our knowledge, this is the first scoping review focused on interventions tested for their impact on mental health issues associated with LC. Seventeen studies were identified, with a wide range of recruitment methods, number of participants recruited, intervention tested, duration of the intervention, practitioners involved in delivering the intervention, outcome measures, and post intervention follow up. Various tools were employed to measure a wide range of outcomes. Results were generally positive, with all included studies reporting improvements in mental health outcomes. However, as most studies were case reports, which are known to be of low-quality evidence compared to RCTs and cohort studies [67], the findings of these studies should be interpreted with caution.

### Recruitment and participants

All participants recruited into the included studies were reported to have LC. However, it was not clear in some studies how long participants had been suffering from LC or when they had their acute COVID-19 infection. These participants may have varied with regard to their LC diagnosis, and the duration of time since they had acute COVID-19 infection. The lack of a consistent definition of LC resulted in a heterogeneous sample of participants joining research at different stages of their illness. This might have implications on the impact of the interventions, and the number of symptoms and symptom severity. Therefore, future research needs to take this into consideration and also interrogating whether interventions have different impacts at the different stages of the LC journey.

The over-representation of women and older participants reflects the literature indicating that these are risk factors for LC [26, 28, 68]. However, some studies indicate that people from minority ethnic backgrounds are more likely to acquire a COVID-19 infection compared to the general population [69–71].

Thus, the lack of ethnic diversity in studies in this review leaves a knowledge gap and suggests particular attention be given to ethnic and cultural diversity in future studies.

### Outcomes and outcome measures

Mental health was assessed in multiple ways, mostly focusing on anxiety and depression, with a number of studies also considering QoL. This reflects the broad nature of mental health and that it encompasses multiple aspects of people’s physical, social and psychological life [72]. A wide range of measures were adopted, making cross-study comparison difficult. Researchers might consider using a standardised core outcome set to enhance the comparability of outcomes in future research, as suggested in a recent Delphi consensus study [73]. Additionally, from a patient and public engagement standpoint [74], people with LC could support future trials throughout the research process, including in selecting the best questionnaires to use for the targeted outcome in manners that resonate with their lived experience. Almost all studies assessed targeted outcomes before and after the intervention, with few studies conducting further follow-up measurements to follow progress over time. The addition of follow-up assessments in future research is of high importance, as this would allow researchers to identify the longer-term impact of their intervention [75].

### Interventions and findings

Although this review included only interventions targeting the mental health of patients with long COVID, a wide range of interventions was described. Some interventions were of specific pharmacologic products or looked at dietary change. However, the majority of studies used an integrated approach to try to address the different elements of participants’ mental health and QoL. Most integrated treatments were delivered by a multidisciplinary team and patients needed to take active roles in implementing agreed therapeutic strategies.

Given the heterogeneity of interventions described, no specific ‘standard’ treatment can be delineated. However, our review suggests that a wide variety of interventions have the potential to support mental health of individuals with LC. and a variety of integrated care models have been examined.

All interventions used by studies in this review were associated with positive outcomes. However, considering study designs of most included studies, the findings should be interpreted cautiously. More large-scale, high-quality trials are needed to define effective intervention strategies for LC. As LC presentation varies between individuals, interventions addressing a wide range of symptoms including mental health and QoL could be promising in building the evidence base.

## Strengths and limitations

The findings of this scoping review contribute to the body of knowledge on LC. Strengths of the review include: 1) Adherence to the PRISMA-ScR (Preferred Reporting Items for Systematic Reviews and Meta-Analyses Extension for Scoping Reviews) guidelines [45], including the widest available evidence through a combination of complementary keywords to search all related databases and was also covering grey literature and the reference lists of all included studies and related reviews; 2) no language restrictions - all retrieved studies in different languages were examined, translated, and included; and 3). Selection bias was minimised by having two independent reviewers conducting the title, abstract and full text screening, with a sample of data extraction being reviewed by a third person.

However, some limitations were encountered with this review. There was missing data from some included studies despite attempts to contact corresponding authors, which precluded rigorous comparisons being made. Another limitation was related to the search strategy, which was limited to studies where mental health was the primary outcome. This may have led to missing some important interventions that may have had positive impacts on mental health as a secondary outcome.

## Conclusions

A wide range of interventions designed to support mental health in patients with long COVID has been examined. Overall, these interventions reported positive effects on mental health outcomes, though more research is needed to confirm this. Considering the ongoing nature of LC and treatment recommendations to date, it is important to address patients’ care needs holistically, ideally through using an integrated care approach.

## Supplementary Information

**Additional file 1. **Example of search strategy using Medline.

## Data Availability

All data generated or analysed during this study are included in this published article.

## Abbreviations

BDI-II: Beck Depression Inventory-II
BAI: Beck Anxiety Inventory
BRS: Brief Resilience Scale
CADTH: Canadian Agency for Drug and Technologies in Health
CBT: Cognitive Behavioural Therapy
DASS-21: Depression, Anxiety and Stress Scale-21
EQ-5D-5L: Euro-Qol 5 dimensions 5 levels
GAD-7: Generalized Anxiety Disorder-7
FAS: Fatigue Assessment Scale
HADS: Hospital Anxiety and Depression Scale
HDRS: Hamilton Depression Rating Scale
LC: Long COVID
NICE: National Institute for Health and Care Excellence
NR: Not reported
PHQ-9: Patient Health Questionnaire 9 items
PRISMA-ScR: Preferred Reporting Items for Systematic Reviews and Meta-Analyses Extension for Scoping Reviews
PSS-10: Perceived Stress Scale-10
RCT: Randomised Controlled Trial
SCIE: Social Care Institute for Excellence
SF-12: 12-Item Short Form Survey
SF-36: 36-Item Short Form Survey
WAM: Wellbeing, Activity and Mood questionnaire
WHHQ-18: Warwick Holistic Health Questionnaire
WHO: World Health Organisation
Zung SRDS: Self-Rating Depression Scale
Zung SRAS: Self-Rating Anxiety Scale
QoL: Quality of life
