# Supplementary material for: Interventions to support mental health in people with long COVID: a scoping review
Source: BMC Public Health. 2023 Jun 20;23:1186. doi: 10.1186/s12889-023-16079-8 (PMC10280822; doi:10.1186/s12889-023-16079-8)
Supplement: Supplementary file 1 — Additional file 1. Example of search strategy using Medline. [file 12889_2023_16079_MOESM1_ESM.docx]

**Additional file 1. Example of search strategy using Medline**

Example of search strategy using Medline

|  | Search |
| --- | --- |
| 1 | (intervention* or treatment* or support* or therap* or service or program*).mp |
| 2 | (mental* or psychiatr* or "post-trauma*" or posttrauma* or PTSD or depress* or anxiet* or dysthymi* or phobia* or panic* or psychopath*).mp |
| 3 | ("long COVID" or "long covid*" or longcovid or "long-COVID" or "post COVID" or "post-acute COVID" or "post acute COVID" or "long haul*" or "COVID sequelae" or "sequelae of COVID" or "post-SARS-COV-2" or PASC or "Post-acute Sequelae of COVID-19").mp |
| 4 | 1 and 2 and 3 |
| 5 | Limit 4 to (journal article) |
| 6 | Limit 5 to dt=20200101-20221003 [January 1^st^, 2020 to October 3^rd^, 2022] |
